# Differences in protein expression, at the basal state and at 2 h of insulin infusion, in muscle biopsies from healthy Arab men with high or low insulin sensitivity measured by hyperinsulinemic euglycemic clamp

**DOI:** 10.3389/fendo.2022.1024832

**Published:** 2023-02-17

**Authors:** Ilham Bettahi, Roopesh Krishnankutty, Morana Jaganjac, Noor Nabeel M. Suleiman, Manjunath Ramanjaneya, Jayakumar Jerobin, Shaimaa Hassoun, Meis Alkasem, Ibrahem Abdelhakam, Ahmad Iskandarani, Tareq A. Samra, Vidya Mohamed-Ali, Abdul Badi Abou-Samra

**Affiliations:** ^1^ Qatar Metabolic Institute, Academic Health System, Hamad Medical Corporation, Doha, Qatar; ^2^ Translational Research Institute, Academic Health System, Hamad Medical Corporation, Doha, Qatar; ^3^ Division of Molecular Medicine, Ruder Boskovic Institute, Zagreb, Croatia; ^4^ Weill Cornell Medicine-Qatar, Doha, Qatar; ^5^ Anti-doping Laboratory, Doha, Qatar

**Keywords:** insulin sensitivity, insulin resistance, HIEC, proteomics, mitochondria, diabetes

## Abstract

**Background:**

Skeletal muscle is the main site for insulin-dependent glucose disposal. The hyperinsulinemic euglycemic clamp (HIEC) is the gold standard for the assessment of insulin sensitivity (IS). We have previously shown that insulin sensitivity, measured by HIEC, varied widely among a group of 60 young healthy men with normoglycemia. The aim of this study was to correlate the proteomic profile of skeletal muscles to insulin sensitivity.

**Methods:**

Muscle biopsies from 16 subjects having the highest (M ≥ 13; *n* = 8, HIS) and lowest (M ¾ 6, *n* = 8, LIS) IS were obtained at baseline and during insulin infusion after stabilization of the blood glucose level and glucose infusion rate at the end of the HIEC. The samples were processed using a quantitative proteomic analysis approach.

**Results:**

At baseline, 924 proteins were identified in the HIS and LIS groups. Among the 924 proteins detected in both groups, three were suppressed and three were increased significantly in the LIS subjects compared with the HIS subjects. Following insulin infusion, 835 proteins were detected in both groups. Among the 835 proteins, two showed differential responsiveness to insulin; ATP5F1 protein was decreased, and MYLK2 was higher in the LIS group compared with that in the HIS group. Our data suggest that alteration in mitochondrial proteins and an increased number of proteins involved in fast-twitch fiber correlate to insulin sensitivity in healthy young Arab men.

**Conclusions:**

These results suggest a change in a small number of differentially expressed proteins. A possible reason for this small change could be our study cohorts representing a homogeneous and healthy population. Additionally, we show differences in protein levels from skeletal muscle in low and high insulin sensitivity groups. Therefore, these differences may represent early events for the development of insulin resistance, pre-diabetes, and type 2 diabetes.

## Introduction

Type 2 diabetes (T2D) represents a significant international health challenge, with approximately 463 million people having diabetes in 2019—half of whom are undiagnosed (1). Over the past few years, the prevalence of T2D has been dramatically increasing in the Middle East and North Africa region, including Qatar (2). Insulin resistance in skeletal muscle is recognized as the earliest metabolic defect in T2D (3). Insulin resistance is a complex heterogeneous phenomenon influenced by genetic and environmental factors (4). Several abnormalities can predict insulin resistance, including impaired insulin activation of glycogen synthase, impairment of the proximal components of insulin signaling (5–7), and increased intramuscular triglyceride content (5). Moreover, insulin-stimulated glucose oxidation and insulin inhibition of lipid oxidation are impaired in subjects with insulin resistance and T2D (6). The inability to switch from lipid to carbohydrate has been described as “metabolic inflexibility” in insulin-resistant subjects (7). Moreover, a reduction in the activity of oxidative enzymatic pathways and dysfunction of the mitochondria have been observed in skeletal muscle obtained from subjects with T2D and correlate with the severity of insulin-resistant glucose metabolism (8). Previous longitudinal studies show that insulin resistance is familial and occurs many years before the development of glucose intolerance (9). Whether genetic or acquired, the resistance of skeletal muscles to insulin may be associated with alteration in the expression of key proteins involved in glucose homeostasis.

Several studies have reported the skeletal muscle proteomic profile from skeletal muscle biopsies of humans and mice (10–14). Hojlund et al. showed that the abundance of certain proteins, such as heat shock proteins, which are altered in skeletal muscles, and key mitochondrial metabolic pathways, such as ATP synthase and creatine kinase B, are perturbed in patients with T2D (10). Hwang et al. demonstrated a reduced abundance of several mitochondrial proteins in the insulin-resistant muscle compared with the healthy group (11). Another study which looked at the mitochondria isolated from insulin-resistant skeletal muscle using one-dimensional gel electrophoreses and high-performance liquid chromatography/electrospray ionization–tandem mass spectrometry (HPLC/ESI–MS/MS) showed a lower abundance of proteins involved in branched-chain amino acid metabolism in T2D than in the lean control (12). Previous studies have shown that proteomic markers of insulin resistance can be determined in T2D subjects. However, these studies have not elucidated if early changes in insulin sensitivity (IS) in healthy people are associated with different protein expression in muscles. Furthermore, protein responses to hyperinsulinemic euglycemic clamp (HIEC) in people with low *versus* high IS have not been shown previously.

We have recently reported that insulin sensitivity, measured by HIEC, varied widely among euglycemic young healthy men (15) and correlated with circulating metabolomic signatures (16). The goal of the present study was to evaluate the altered expression pattern of skeletal muscle proteins associated with reduced insulin sensitivity in muscle biopsies taken at the basal state and during insulin infusion, at the end of the insulin clamp, when both glycemia and glucose infusion have stabilized. We used advanced proteomic techniques to identify a unique list of candidate proteins both at baseline and during insulin infusion, allowing identification of the proteins that correlate with insulin sensitivity, which may provide further information as to the molecular mechanisms of reduced insulin sensitivity in apparently healthy euglycemic subjects.

## Materials and methods

### Study participants

**Figure 1:**
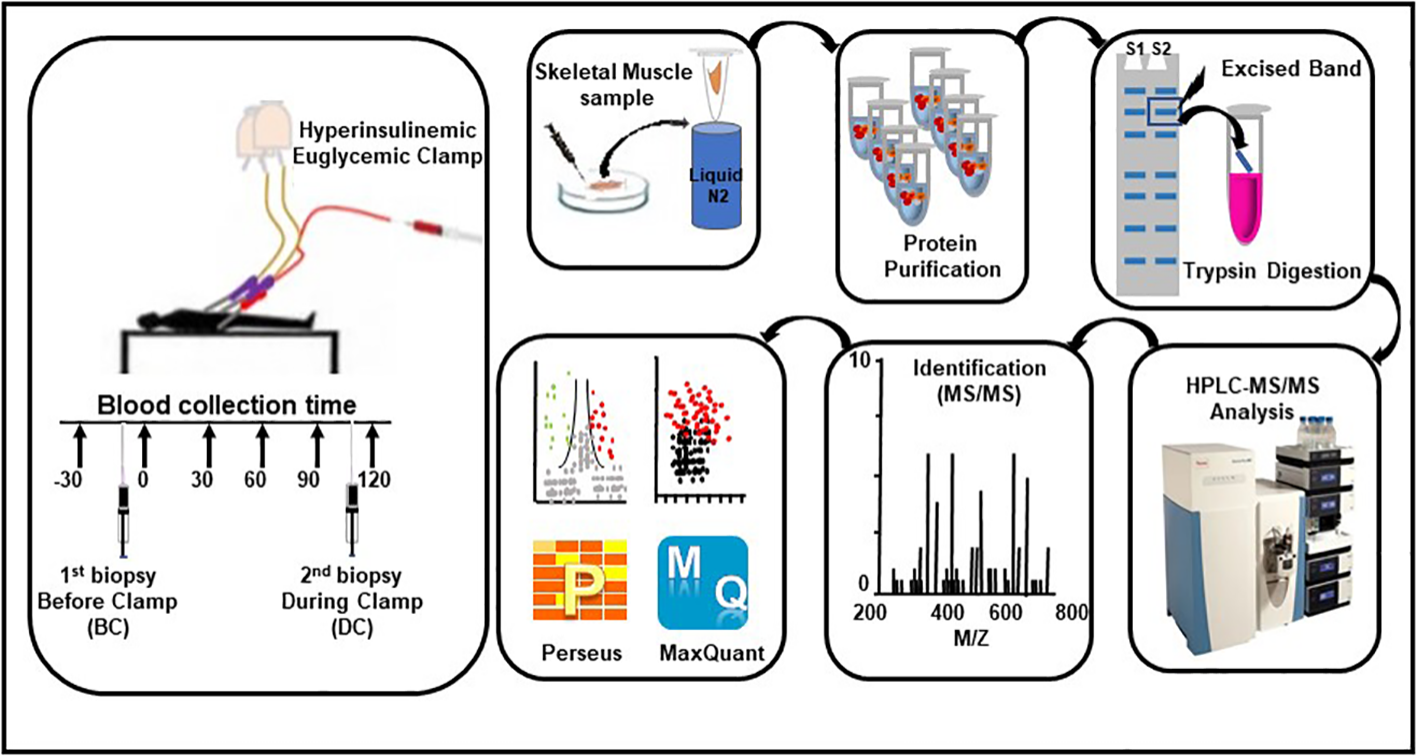
Workflow for the hyperinsulinemic euglycemic clamp, skeletal muscle biopsies, sample processing, and proteomic analysis.

The overall design of the study flow is summarized in **Figure 1**. The details on the subjects and study protocol were previously reported (15). In brief, healthy young men of Arab descent (*n* = 60) were examined for insulin sensitivity using HIEC. Muscle biopsies were obtained from the vastis lateralis before the clamp and during insulin infusion at the end of the clamp, when the plasma glucose level and the glucose infusion rate were stabilized (15). The study was approved by the Institutional Review Board protocol (14224/14) of Hamad Medical Corporation, Doha, Qatar. All participants gave their signed informed consent. Participants were included in the study if they satisfied all the following criteria (1): age >18–45 years, (2) body mass index ≤28 and ≥16, (3) normal CBC, (4) normal blood chemistry, (5) normal fasting glucose, (6) normal HbA1c, (7) normal glucose response to 75 g oral glucose tolerance test performed after 8 h of fasting, (8) normal ECG, and (9) commitment to the whole study protocol. Proteomic analyses were performed on muscle biopsies obtained from eight subjects who showed the highest insulin sensitivity (HIS) and eight other subjects who showed the lowest insulin sensitivity (LIS) (**Table 1**) among the 60 subjects reported previously (15).

**Table 1:** Anthropometric and metabolic characteristics of the subjects with low insulin sensitivity (LIS) and high insulin sensitivity (HIS).

	High insulin sensitivity (*N* = 8)	Low insulinsensitivity (*N* = 8)	*p*-value
Age (year)	31.5 ± 6.0	33.1 ± 6.0	0.23
BMI (kg/m^2^)	26.2 ± 2.1	24.7 ± 2.1	0.12
M (mg/kg/min)	17.0 ± 1.9	5.3 ± 1.0	0.00*
Fasting glucose (mmol/L)	5.54 ± 0.39	5.25 ± 0.20	0.09
2 h glucose (mmol/L)	5.39 ± 1.08	5.10 ± 0.50	0.53
HB1A1C %	5.2 ± 0.2	5.2 ± 0.2	0.86
TG (mmol/L)	0.86 ± 0.41	1.06 ± 0.41	0.39
Total cholesterol (mmol/L)	4.50 ± 0.93	5.15 ± 1.23	0.37
HDL cholesterol (mmol/L)	1.23 ± 0.30	1.31 ± 0.26	0.62
LDL cholesterol (mmol/L)	2.86 ± 0.73	3.37 ± 1.01	0.40

BMI, body mass index; HB1A1C, hemoglobin A1C; TG, triglyceride; HDL, high-density lipoprotein; LDL, low-density lipoprotein.

Average ± SD. *p < 0.05.

### Hyperinsulinemic euglycemic clamp

As previously reported (16), the subjects were admitted to the research study unit at 7 a.m., after 10–12 h of overnight fasting, and a baseline muscle biopsy was obtained before the clamp study. Three polyethylene catheters were inserted in the antecubital fossa and back of the hand veins, enabling insulin/dextrose infusions, blood glucose measurements, and blood sampling. The insulin infusion (100 IU/ml insulin solution, Actrapid) rate was constant throughout the HIEC [40 mU/body surface area (m^2^)/min]. The body surface area (m^2^) [0.007184 x (height(cm)^0.725^) x (weight(kg)^0.425^)] was calculated as described. The blood glucose level was modulated by the infusion of 20% dextrose, which was adjusted every 5 min to achieve a blood glucose level of 90 mg/dl (5 mmol/L). A second muscle biopsy was obtained under insulin infusion at 120 min (after the glucose infusion was stabilized). Similar to previous studies, the duration of the HIEC procedure was 120 min (17–19). Insulin sensitivity, as reflected by the whole-body glucose disposal rate (M-value, milligram of glucose infused per kilogram of body weight per minute), was computed after the stabilization of glycemia and of infusion rate during the last 60 min of the euglycemic clamp; this showed a wide variation ranging from 2 to 20 (16). Muscle biopsies were quick-frozen in liquid nitrogen. In this study, we selected only the subjects with the lowest and the highest insulin sensitivities for proteomic analyses; the size of the groups was based on previous human studies (20–23).

### Sample preparation for proteomic assay

The frozen muscle biopsy samples of individuals with LIS (M ≤6, *n* = 8) and HIS (M >13, *n* = 8) were ground into fine powders using a mortar and pestle and liquid nitrogen. Protein extracts were isolated from the tissue samples using RIPA buffer. The lysates were centrifuged at 15,000 rpm for 10 min at +4°C, and the supernatants were transferred to new tubes, with the protein concentration determined using a BCA Protein Assay Kit (Pierce). The normalized protein samples were electrophoretically separated on 10% SDS-PAGE; whole lines were excised and divided into eight equal parts, as previously described (24). Gel pieces were then reduced with 10 mM dithiothreitol in 25 mM ammonium bicarbonate and alkylated with 10 mM iodoacetamide in 25 mM ammonium bicarbonate, followed by overnight digestion at 37°C using 20 ng/µl Trypsin/Lys-C (Promega). The peptides were eluted using 1% formic acid, and the volume was reduced to 20 μl using a vacuum centrifuge (Eppendorf, Hamburg, Germany).

### Liquid chromatography–tandem mass spectrometry

Complex peptide mixtures were analyzed by shotgun proteomics using an Easy n-LC II (Thermo Scientific, Waltham, MA, USA) coupled to an Orbitrap Elite mass spectrometer (Thermo Scientific, Waltham, MA, USA), as previously described (24).

### MS data processing and statistical analysis

MS data processing and analysis were performed according to Leo et al. in 2019 (25). The MaxQuant software version 1.6.17.0, according to the standard workflow with the built-in search engine Andromeda using the Uniprot human reference proteome database (downloaded October 12, 2017), was used for protein identification. The Max label-free quantification (LFQ) method, with retention time alignment and match-between-runs features in MaxQuant, was applied to extract the maximum possible quantification information. Protein abundance was calculated based on normalized spectral intensity (LFQ intensity).

MS data analysis was performed using the open-source software Perseus (version 1.6.14.0) (26). The protein quantification and the statistical significance between the two groups were calculated using two-tailed *t*-test with permutation-based false discovery rates (FDR) of at least ±1.5 fold (*p* < 0.05). Functional enrichment analysis of the differentially abundant proteins was carried out using the online bioinformatics resource Database for Annotation, Visualization, and Integrated Discovery (DAVID). The distribution of proteins enriched under different categories, such as cellular components, biological processes, and pathways including Kyoto Encyclopedia of Genes and Genomes (KEGG) and Reactome, was identified. Proteomic analysis was performed to identify proteins differentially expressed between baseline, before clamp (BC), and under insulin infusion during clamp (DC) as well as between LIS and HIS subjects both at baseline and under insulin infusion. The differences in baseline demographic, clinical, and biochemical data between LIS and HIS as presented in **Table 1** were assessed by *t*-test.

### Functional annotation and pathway identification

Functional annotation was performed by gene set enrichment analysis using g:Profiler (27). The statistically significant enrichment of biological processes and KEGG and Reactome pathways is extracted and plotted (27) using proteins that are uniquely expressed or significantly altered (increased or decreased) in HIS *versus* LIS at baseline or during insulin stimulation.

## Results

### Effects of insulin infusion on protein expression in the muscle biopsies

**Figure 2:**
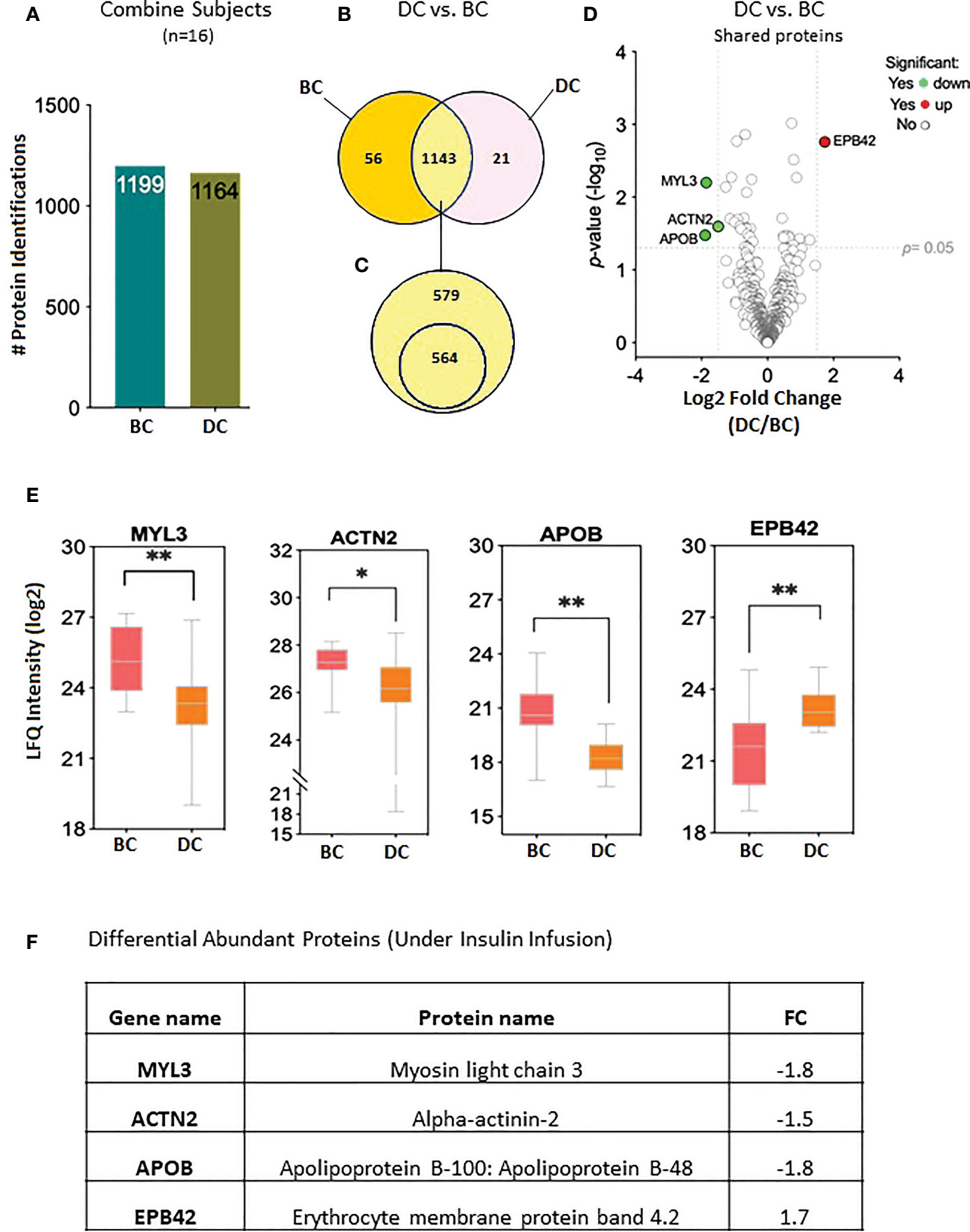
Proteins levels at the baseline and at 2 hours of insulin infusion in the 16 subjects. **(A)** Bar chart representing the number of proteins identified in each condition. **(B)** Venn diagram showing the unique and common proteins in response to clamp. **(C)** Proteins sorted for their presence in at least half of the subjects. **(D)** Volcano plot showing the differential abundance of proteins in response to insulin infusion. **(E)** Box plots representing the proteins with significant differential abundance. *p=0.02, **p=0.001. BC: Before Clamp (basal values); DC: During Clamp (insulin infusion for 2 hours). **(F)** Fold changes in the proteins significantly increased during insulin stimulation versus baseline".

The proteomic profiles of all 16 subjects were analyzed to study the protein expression pattern between baseline (BC) and under insulin stimulation DC. The proteomic analysis resulted in the identification of 1,199 proteins BC and 1,164 proteins under insulin stimulation DC (**Figure 2A**); 56 proteins were only present in subjects before clamp and 21 proteins were present only under insulin stimulation; 1,143 proteins were present both at basal condition and under insulin stimulation (**Figure 2B**). Out of the 1,143 shared proteins, 564 were present in at least 50% of the subjects (**Figure 2C**; **Supplementary Table S1**). Among the 1,143 shared proteins, four are differentially expressed—one protein was increased and three were reduced under insulin stimulation. The significant difference in protein abundance in response to insulin after comparative proteome analysis is graphically represented as a volcano plot, drawn using the fold change and the *p*-value (**Figure 2D**). The LFQ of the proteins with significant differential abundance is shown in **Figure 2E** and the fold changes in **Figure 2F**.

### Differences in protein expression in muscle biopsies from subjects with low and high insulin sensitivities at baseline

**Figure 3:**
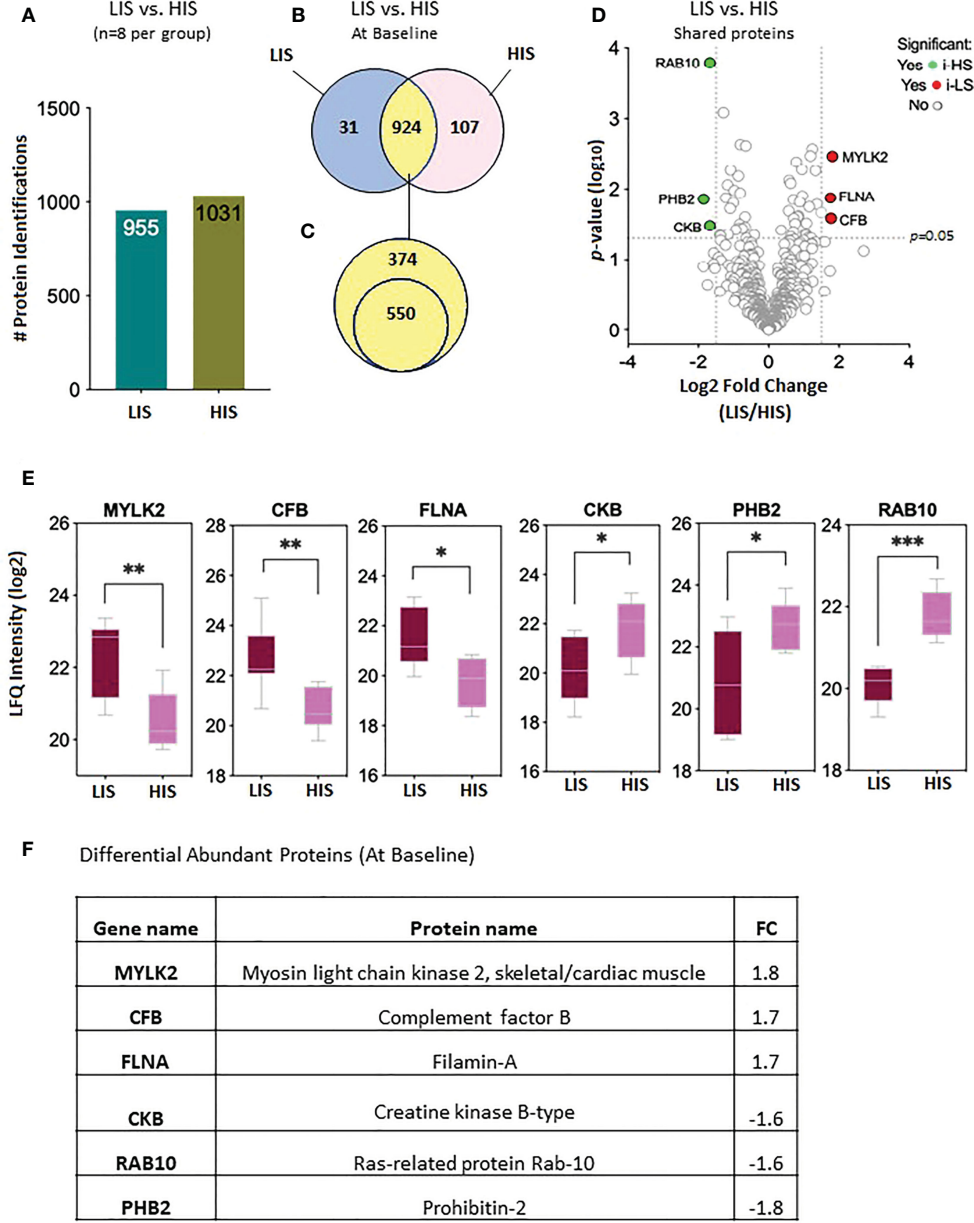
Baseline protein levels in the subjects with low insulin sensitivity (LIS) versus the subjects with high insulin sensitivity (HIS). **(A)** Bar chart representing the number of proteins identified in each group. **(B)** Venn diagram showing the unique and common proteins in LIS and HIS. **(C)** Proteins sorted for presence in at least 50% of the subjects. **(D)**. Volcano plot showing the differential abundance of proteins present in both the groups. **(E)** Box plots representing the proteins with significant differential abundance. *p=0.02, **p=0.002, *** p=0.0003. **(F)** Fold changes in the proteins showing statistically significant differential abundance in LIS versus HIS at baseline.

The proteomic data were analyzed to identify differential protein expression at baseline (before clamp) between the LIS and HIS subjects. This analysis identified a total of 1,986 proteins (**Figure 3A**) that were reduced to 1,062 different proteins (**Figure 3B**). After sorting, 31 unique proteins were detected in the LIS group, 107 unique proteins were detected in the HIS group, and 924 proteins were detected in both groups (**Figure 3B**). Out of the 924 proteins shared between the two groups, 550 proteins were found in at least 50% of the subjects of both groups (**Figure 3C**; **Supplementary Table S2**). Among the shared proteins, six proteins had differential abundance—of which three showed a higher expression and three a lower expression in HIS *versus* LIS. The significant difference between the groups from the comparative proteome analysis is graphically represented as a volcano plot, drawn using the fold change and the *p*-value (**Figure 3D**). The LFQ of the proteins with significant differential abundance is shown in **Figure 3E** and the fold changes in **Figure 3F**. One of the differentially abundant proteins (complement factor B) was identified as a biomarker for diabetes after sorting against the list of proteins identified to be involved in diabetes, as indicated in the peptide atlas database (28).

### Differentially enriched pathways in relation to insulin sensitivity status [LIS *versus* HIS subjects at baseline (“baseline insulin sensitivity–enriched pathway”)]

**Figure 4:**
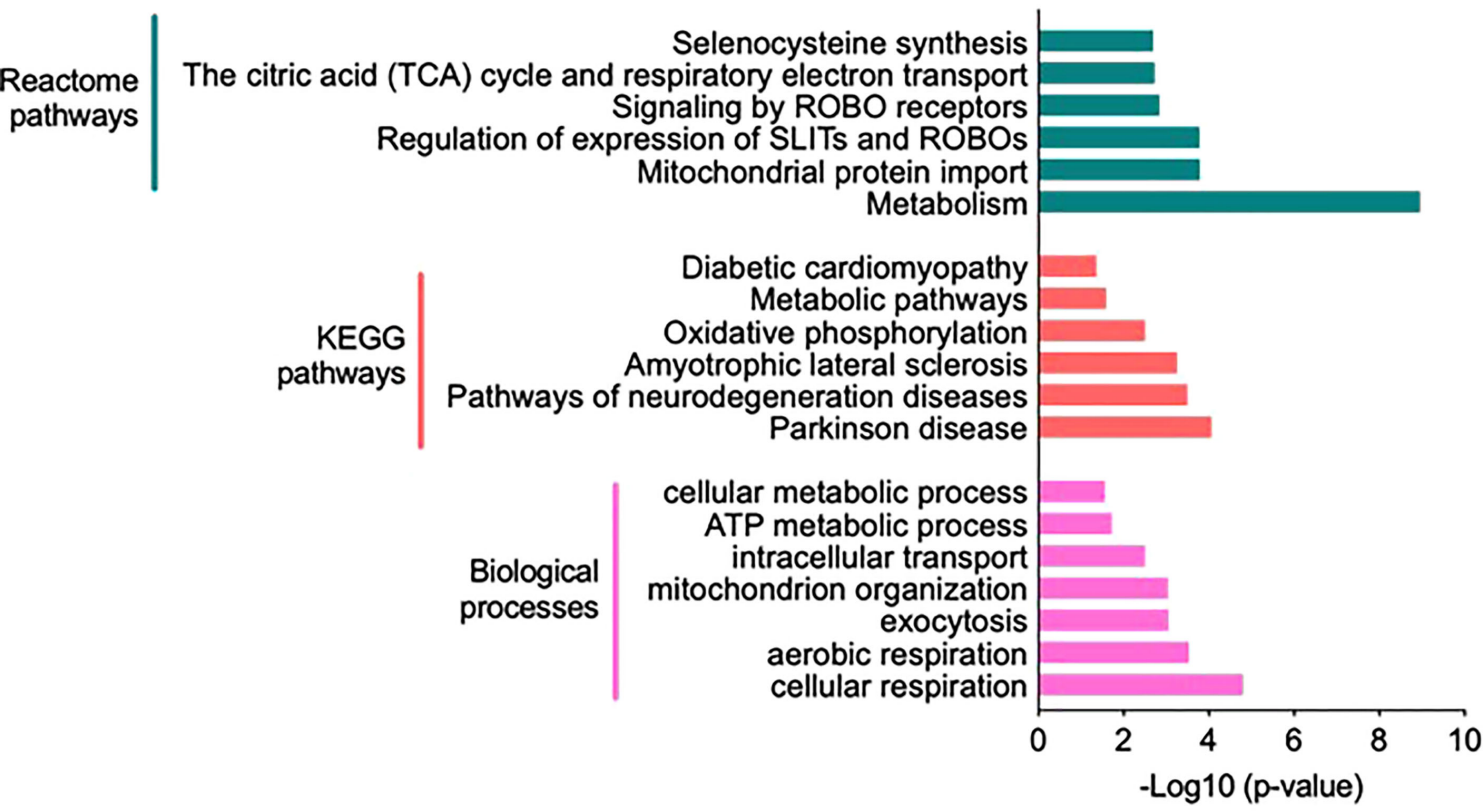
Functional annotation and classification by enrichment analysis of proteins uniquely present in the high insulin sensitivity group. Top enriched terms and their distribution categorized into biological processes, Kyoto Encyclopedia of Genes and Genomes pathways, and Reactome pathways (**Supplementary Table S6**).

Significantly altered biological processes resulted from 107 proteins uniquely expressed in the HIS group (**Figure 3B**), such as cellular metabolic process, intracellular transport, and aerobic/cellular respiration. The most enriched terms under the category are shown in **Figure 4**. Among the KEGG pathways, diabetic cardiomyopathy, metabolic pathways, and oxidative phosphorylation were found to be the most enriched pathways (**Figure 4**; **Supplementary Table S6**). Functional enrichment analysis using unique proteins also identified selenocysteine synthesis, citric acid (TCA) cycle, signaling by ROBO receptors, mitochondrial protein import, and metabolism as the pathways active in patients with high insulin sensitivity, as these were the most enriched terms under the Reactome pathways (**Figure 4**; **Supplementary Table S6**), while no significant enrichment in any of these pathways was observed in the low insulin sensitivity group.

### Effects of insulin infusion on protein expression in the muscle biopsies from subjects with low *versus* high insulin sensitivity

**Figure 5:**
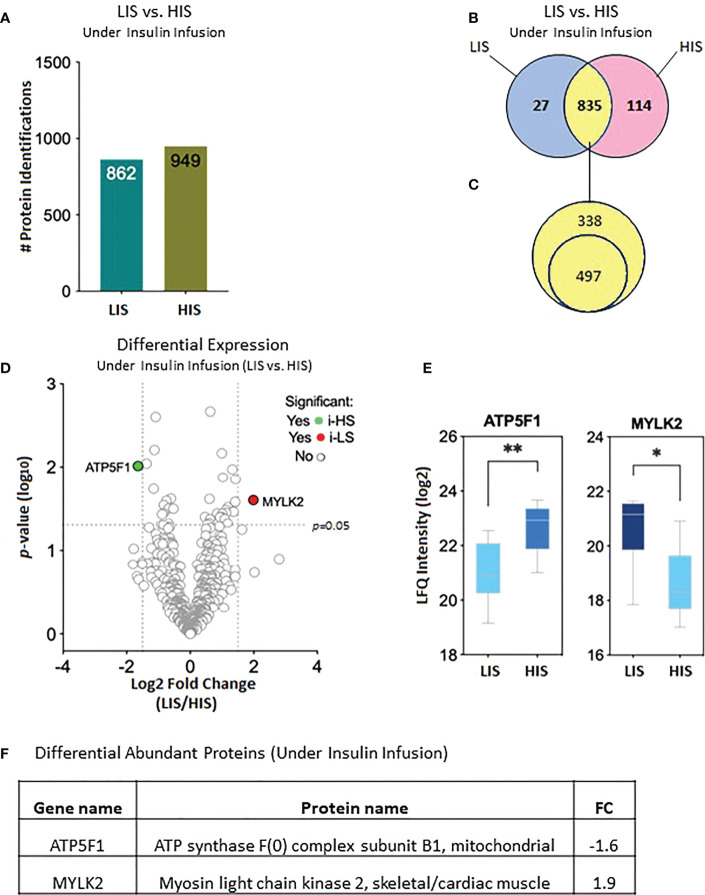
Effects of insulin (2 h infusion) on protein levels in the subjects with low insulin sensitivity (LIS) versus the subjects with high insulin sensitivity (HIS). **(A)** Bar chart representing the number of proteins identified in each group. **(B)** Venn diagram showing the unique and common proteins in response to clamp. **(C)** Proteins sorted for their presence in at least 50% of the subjects. **(D)**. Volcano plot showing the differential abundance of proteins between the groups after 2 hours of insulin infusion during the HIEC. **(E)** Box plots representing the proteins with a significant differential abundance. *p=0.01, **p=0.001. **(F)** Fold changes in the proteins showing statistically significant differential abundance in LIS versus HIS under-insulin stimulation.

The proteomic data of the LIS and HIS subjects under insulin infusion were analyzed to identify proteins that differ in their response to insulin between the two groups. After combining all data, 862 proteins were identified in the LIS group and 949 proteins in the HIS group, assigned to be exclusively present in at least one of the eight subjects per group, respectively (**Figure 5A**). After sorting, 27 proteins were found to be uniquely present in the LIS group and 114 proteins in the HIS group, while 835 proteins were shared between the two groups (**Figure 5B**). Out of the 835 proteins shared between the two groups, 497 proteins were found to be present in at least 50% of the subjects of both groups (**Figure 5C**; **Supplementary Table S3**). Two proteins show a significant difference (**Figures 5D, E**). The comparative proteome analysis of shared proteins is graphically represented as a volcano plot, drawn using the fold change and the *p*-value (**Figure 5D**), LFQ (**Figure 5E**), and fold change (**Figure 5F**).

### Pathways differentially enriched in response to insulin infusion subjects with low and high insulin sensitivities

**Figure 6:**
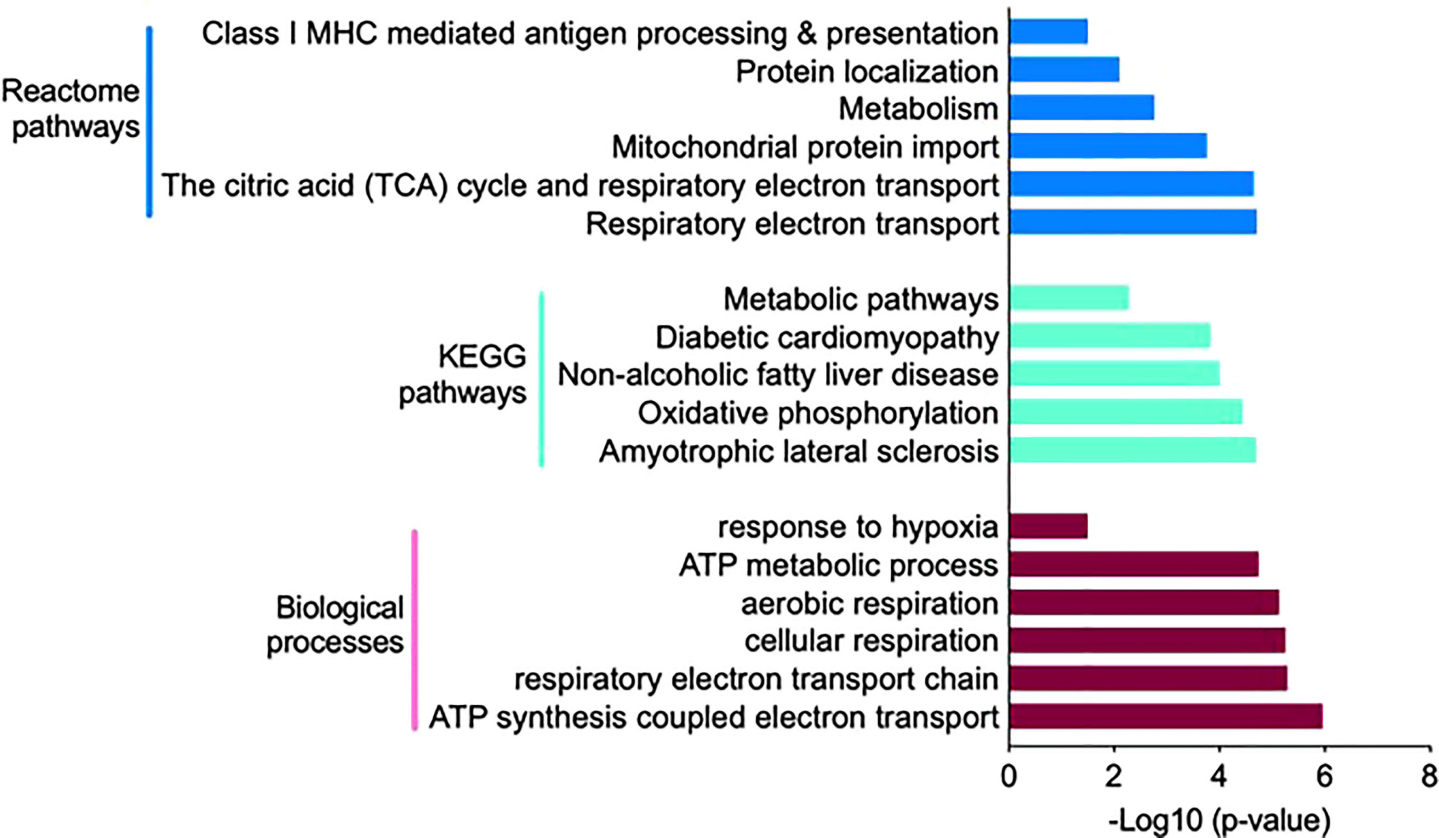
Functional annotation and classification by enrichment analysis of proteins uniquely present in HIS or LIS group and those showing differential abundance. The top enriched terms and their distribution categorized into biological processes, KEGG pathways and Reactome pathways.

The 114 proteins (**Figure 5B**) uniquely present in HIS subjects in response to insulin infusion were found to be involved in biological processes such as response to hypoxia, ATP metabolic process, aerobic and cellular respiration, and respiratory electron transport chain as these were the most enriched terms under the category (**Figure 6**; **Supplementary Table S7**). Among the KEGG pathways, metabolic pathways, diabetic cardiomyopathy, non-alcoholic fatty liver disease, and oxidative phosphorylation were found to be the most enriched terms (**Figure 6**; **Supplementary Table S7**). Functional enrichment analysis using unique proteins also identified class I MHC-mediated antigen processing and presentation, protein localization, metabolism TCA cycle, and respiratory electron transport as the pathways active in high insulin sensitivity patients in response to insulin clamp, as these were the most enriched terms under the Reactome pathways (**Figure 6**; **Supplementary Table S7**), while no significant enrichment in any of the pathways was observed in the LIS group.

### Effects of insulin infusion on protein expression in the muscle biopsies from each group (LIS and HIS)

The differentially abundant proteins in response to insulin stimulation were analyzed in each group (LIS and HIS). Only one protein was found to be increased, while six proteins were identified to be decreased in response to the clamp in the LIS group (**Table 2**; **Supplementary Table S4**). In the case of the HIS group, three proteins were increased in response to the clamp, while only two proteins were found to be decreased (**Table 2**; **Supplementary Table S5**).

**Table 2:** Proteins showing the highest response to insulin stimulation statistically significant (p value at least <0.05) differential abundance (more than 1.5 fold) among 16 volunteers between the subjects with low insulin sensitivity (LIS) and high insulin sensitivity (HIS) both at base line and after insulin stimulation.

Gene name	Protein name	Fold change	Group
MYL1	Myosin light chain 1/3, skeletal muscle isoform	−2.0	Low insulin sensitivity
APOB	Apolipoprotein B-100	−3.5	
UBE2V2	Ubiquitin-conjugating enzyme E2 variant 2	−1.8	
MYL3	Myosin light chain 3	−2.9	
NDUFS2	NADH dehydrogenase (ubiquinone) iron–sulfur protein 2, mitochondrial	−2.1	
CENPF	Centromere protein F	−1.9	
BCAM	Basal cell adhesion molecule	1.7	
GRB2	Growth factor receptor-bound protein 2	−1.6	High insulin sensitivity
NDUFS5	NADH dehydrogenase (ubiquinone) iron–sulfur protein 5	−2.2	
EPB42	Erythrocyte membrane protein band 4.2	1.6	
PLG	Plasminogen	1.5	
CFH	Complement factor H	1.8	

The data are fold changes (FC) comparing level of protein under insulin stimulation during the hyper insulinemic euglycemic clamp versus baseline.

## Discussion

Skeletal muscles, liver, and fat are the main insulin target tissues; however, muscles play a major role in glucose clearance under insulin stimulation and strongly correlate with whole body insulin sensitivity (29). We have previously shown that whole body sensitivity to insulin, measured by a hyperinsulinemic euglycemic clamp, varies widely among healthy young men (15). We therefore hypothesized that the skeletal muscle proteome profile may show variations between high and low insulin sensitivity subjects (HIS and LIS). At the basal state, multiple proteins were uniquely detected in the HIS and LIS subjects, and over 85% of total proteins were commonly detected in the two groups. Few of them showed differential expression levels. Based on Gene Ontology using DAVID software, the most abundant unique proteins present in the HIS group before and during insulin infusion showed a significant enrichment of the biological pathways involved in the mitochondria function and TCA and β-oxidation cycle (**Figures 4** **–** **6**).

Under fasting conditions, three common proteins are significantly downregulated, and three others are upregulated in the LIS group compared with the HIS group [*p* < 0.05, −1.5 ≥ fold change (FC) ≥1.5, FDR <0.05%]; these were involved in mitochondria function, Glut4 translocation, and structural and contractile proteins. Interestingly, we observed a significant downregulation of creatine kinase B (CKB) as a marker for anaerobic ATP resynthesis enzyme in the LIS group compared with the HIS group. Consistent with our study, Højlund et al. showed that, in the human skeletal muscle, the level of CKB was reduced in T2D (10). CKB may play a specific role in mitochondrial fuel oxidation (10, 30). A significant observation of our analysis is that the Ras-related protein Rab10 showed a low abundance in 50% of the LIS group. It is well established that the insulin activation of protein kinase B (also known as AKT) leads to the stimulation of the GTP-bound Ras-related protein (Rab10) and thereby triggers GLUT4 vesicle movement to the membrane (31). In addition to Glut4 translocation, Rab-GAP was shown to control the uptake of saturated and unsaturated fatty acid into the skeletal muscle (32). Several studies showed the dysregulation of intramyocellular fatty acid metabolism in the offspring of patients with T2D and obese patients with T2D. Recently, our group performed a metabolomic analysis of circulating plasma metabolites from the same cohort, and found that molecules involved in lipid metabolism, predominantly fatty acids, were upregulated in the LIS group compared with the HIS group (16). A previous study in insulin-resistant muscle revealed reduced protein expression to be involved in mitochondrial function (33). Hence, our results support that the abundance of proteins involved in mitochondrial functions is also downregulated (34). Prohibitin 2 (PHB2), which represents the integrity of the mitochondrial inner membrane (35), was significantly reduced in LIS. The deletion of PHB2 results in the dysfunction of the mitochondria (36). Additionally, at fasting conditions, we found several slightly downregulated proteins (**Supplementary Table S2**) that were involved in mitochondria function and TCA cycle, such as cytochrome b-c1 complex subunit (UQCRFS1; complex III), cytochrome c1 (CYC1; complex II), NADH dehydrogenase (NDUFS3; complex I), and ATP synthase subunit O, mitochondrial (ATP5O; complex V) (**Supplementary Table S2**). In fact, proteins involved in the TCA cycle (37, 38) and ATP synthase (39) tend to be less abundant in the insulin resistance of skeletal muscle.

Mitochondria are the intracellular sites of skeletal muscle fuel oxidation and ATP production, and mitochondrial dysfunction may play a critical role in impaired glucose metabolism observed in the skeletal muscle of T2D patients and their insulin-resistant offspring (40). Furthermore, under insulin stimulation, proteins involved in mitochondrial energy metabolism were downregulated in the LIS group compared with the HIS group. We observed a significantly decreased ATP5F1 protein related to mitochondria synthase, involved in the respiratory chain protein (complex V) complex. Consistent with our finding of altered mitochondria function, most human studies showed mitochondrial dysfunction in skeletal muscle from insulin-resistant offspring of patients with T2D (10, 39, 41, 42), obesity, and T2D (11, 33, 43). Indeed, it has been shown that in skeletal muscle, mitochondrial ATP and mRNA levels and protein synthesis are responsive to insulin infusion in nondiabetic subjects (40); this indicates that insulin signaling modulates certain pathways which may influence mitochondrial proteins and functions. In our proteomic analysis, we observed a slightly low abundance of several proteins involved in mitochondria function in subjects with LIS compared with those in the HIS group, such as ATPC1 (complex V) (FC ≤-1.1) (**Supplementary Table S3**). Yang et al. showed that ATP5C1 is reduced in insulin-resistant non-diabetic Pima Indians (44). Using magnetic resonance spectroscopy, several studies showed that the ATP synthesis rates were lower in the insulin-resistant offspring of T2D patients (39, 41). Hojlund et al. demonstrated a decreased content of the ATP synthase subunit in the skeletal muscles of T2D patients (10). Taken together, these data indicate that the insulin-stimulated rates of ATP synthesis are negatively affected very early in the pathogenesis of insulin resistance (39, 45, 46) and in T2D (47). The current study also reveals alteration in proteins involved in the TCA cycle, such as succinate dehydrogenase (FC ≤-1.2; **Supplementary Table S3**). The SDH complex plays a vital role in cell metabolism, considering its participation in the TCA cycle and the electron transport chain. Sreekumar et al. showed a decreased SDHB expression in skeletal muscle after insulin treatment in T2D patients (48). He et al. also showed that, within each type of fiber, skeletal muscle from obese and T2D had a lower SDH oxidative enzyme activity and increased lipid content compared with those of lean subjects (49). Our data are consistent with the finding of He et al. who showed decreased oxidative enzyme activity and unchanged glycolysis in the skeletal muscle of T2D patients (49).

Human skeletal muscles are constituted of three major fiber types: type 1 (slow oxidative), 2A (fast oxidative glycolytic), and 2X fibers (fast glycolytic), defined by the presence of MYH7 (myosin heavy chain 7), MYH2, and MYH1, respectively. In the fasting state, our analysis showed an upregulation of MYH2 (**Supplementary Table S2**) and myosin light chain kinase 2 (MYLK2) in the LIS group compared with the HIS group; MYLK2 was also significantly upregulated by insulin in the LIS group (**Figures 3E, F**). The MYLK2 gene encodes the skeletal muscle myosin light chain kinase, with higher expression in fast skeletal muscles than in slow muscles. MYLK2 is linked to fast muscle proteins such as myosin light chain 1 (MYL1) (50). At baseline, Giebelstein et al. reported that the upregulation of fast-muscle proteins negatively correlates with insulin sensitivity (33). A study on fiber proportion in human skeletal muscle showed an increase of type 2A fiber (twofold) compared with type 1 in metabolic syndrome subjects.

Another important finding of our analysis is that perilipin 4 (PLIN4) was slightly upregulated under insulin stimulation (FC ≥1.4, **Supplementary Table S3**) in the LIS group compared with the HIS group. Perilipin 4 is expressed in skeletal muscle, heart, and adipose tissues, and it is preferentially located in lipid droplets containing cholesterol ester (51). PLIN4 is recruited to the lipid droplet during droplet formation (52). Poureymour et al. showed that PLIN4 is localized to intramuscular adipocytes and more highly expressed in slow-twitch muscle fibers compared with fast-twitch muscle (52). PLIN4 mRNA is expressed in vastus lateralis biopsies from a healthy individual, and its levels are higher in slow-twitch than fast-twitch muscles. Unlike the PLIN3 protein, PLIN4 expression is reduced in response to prolonged endurance training (53). These data are supported by a previous study that showed an increased intra-myocyte triglyceride level in insulin-resistant first-degree relatives of individuals with T2D (54). Accumulation of intra-myocellular lipid is associated with reduced insulin sensitivity (55).

Moreover, several proteins were altered in the LIS group during insulin infusion compared with the baseline (**Table 2**; **Supplementary Table S4**). Those proteins, the sarcomere proteins myosin light chain1 (MYL1) and myosin light chain 3 (MYL3), were downregulated under insulin stimulation; this is consistent with a previous study that showed downregulation of the slow myosin light chain isoform protein in T2D patients (56). Interestingly, our proteomic analysis revealed (**Table 2**; **Supplementary Table S4**) the downregulation of NDUFS2 (complex I) in the LIS group by insulin; complex I is involved in mitochondrion respiration. Insulin infusion also downregulated apolipoprotein B (APOB); APOB is an important component of LDL and VLDL, which distribute fat molecules to peripheral tissues such as skeletal muscle tissues (57). Excess VLDL secretion has been indicated to deliver increased fatty acids and triglycerides to muscle and other tissues, further inducing insulin resistance (34). Other proteins were altered following insulin stimulation in the LIS group compared to a baseline, such as Centromere protein F, which is involved in skeletal myogenesis, and basal cell adhesion molecule, which is involved in intracellular signaling. Some proteins identified in the HIS group following insulin infusion were different from those in the LIS group (**Table 2**; **Supplementary Table S5**), such as erythrocyte surface protein band 4.2, plasminogen, complement factor H, growth factor receptor-bound protein 2, and NADH dehydrogenase. Interestingly, our study identified a number of proteins involved in the mitochondrion respiratory chain, which were slightly altered in the LIS group compared with the HIS group, including complex I, II, III, and V at fasting condition (**Supplementary Table S2**), whereas following insulin infusion, we detected only two proteins that were altered, one in complex V and the other in TCA. Moreover, our study found that MYLK2 was upregulated in the LIS group compared with the HIS group in both conditions.

The strength of this study was the homogenous representative population of men with normal glycemia levels. Muscle biopsies from the same patients in whom circulating metabolites were measured were also used for proteomic analysis. A limitation of the study is the low number of subjects. Although we screened many participants, most of them failed to meet the criteria for participation in our study. Another limitation of the present study is that biopsies at later time points were not obtained; thus, we may have missed several protein changes that might have occurred at later times, particularly for proteins with a long half-life. Further, obtaining a skeletal muscle biopsy is a complex process and is very difficult to do in a large-scale population, and obtaining multiple timepoint samples is very challenging.

In conclusion, we have demonstrated that human skeletal muscles in apparently healthy male subjects of Arab descent show changes in a small number of proteins related to insulin sensitivity levels. In the fasting state, we found that 12 proteins were differentially expressed in the LIS group compared with the HIS group. Under insulin stimulation, a number of proteins, such as myosin chain and mitochondrial ATP synthase, remained altered in the LIS group. However, we did not detect any changes in glycolytic proteins in both conditions, as also shown in previous studies (11, 58). Collectively, these data provide novel information regarding the metabolic pathways that correlate with insulin sensitivity levels in skeletal muscle and may represent early events for developing insulin resistance, pre-diabetes, and type 2 diabetes.

## Data availability statement

The data presented in the study are deposited in the Figshare repoository, accession number 10.6084/m9.figshare.21988487.

## Ethics statement

Protocols were approved by Institutional Review Boards of the Hamad Medical Corporation, Qatar (IRB protocol #14224/14). All study participants gave their written informed consent prior to participation in the study.

## Author contributions

AA-S designed the study protocol and obtained institutional review board approval. IA, MA, and SH recruited and screened the study subjects and performed the OGTT and HIEC procedures. MJ performed liquid chromatography–MS/MS analyses and provided raw proteomic data. RK performed bioinformatic and statistical analysis of the raw protein data provided by RK. AI, TS, MR, MA, IB, and JJ performed various assay measurements. IB, RK, and AA-S wrote and revised the manuscript. All authors contributed to the article and approved the submitted version.
